# Toll like receptor7 polymorphisms in relation to disease susceptibility and progression in Chinese patients with chronic HBV infection

**DOI:** 10.1038/s41598-017-12698-5

**Published:** 2017-09-29

**Authors:** Junping Zhu, Tong Zhang, Lina Cao, Aixin Li, Kai Zheng, Nan Zhang, Bin Su, Zhiyun Chen, Ning Chen, Hao Wu, Qiushui He

**Affiliations:** 1 Department of Medical Microbiology and Research Centre of Microbiome, Capital Medical University, Beijing, 100069 China; 2 Department of Infectious Diseases, Beijing You’an Hospital, Capital Medical University, Beijing, 100069 China; 3 Department of Medical Microbiology and Immunology, University of Turku, Turku, 20520 Finland

## Abstract

Toll-like receptors (TLRs) play a key role in innate and adaptive immunity, protecting the host from viral pathogens. We studied the effect of TLR7 polymorphisms on disease susceptibility and progression of chronic hepatitis B (CHB) infection in Chinese adults. Blood samples were taken from 612 patients with confirmed CHB, hepatitis B virus (HBV)-related liver cirrhosis (LC) or hepatocellular carcinoma (HCC) and 293 controls. TLR7 polymorphisms (rs179010-C > T, rs2074109-T > C, and rs179009-A > G) were analyzed by PCR-based sequencing. A significantly higher frequency of TLR7 rs179010 C allele was found in male CHB patients than in controls (74.8% vs 59.5%, P = 0.002). The frequency of rs179009 G allele was markedly increased with disease progression when male patients with CHB, LC and HCC were compared (P = 0.012). The haplotype CTA was significantly associated with an increased susceptibility to CHB among male patients (P = 0.000). Frequency of the haplotype CTG was higher in male patients with HCC than CHB (P = 0.005). No such differences in these allele frequencies were found between female patients and controls. Our results indicated that TLR7 polymorphisms play an important role in disease susceptibility and the progression of CHB infections in Chinese adults, and may partly explain the high incidence of HBV related diseases in Chinese men.

## Introduction

Hepatitis B virus (HBV) infection is a global health problem^1^. HBV carriers are at an increased risk of liver damage and many of them suffer from progressive liver diseases, such as chronic hepatitis B infection (CHB), liver cirrhosis (LC) and hepatocellular carcinoma (HCC)^2^. In China, there are 97 million HBV carriers and at least 20 million of them have an active CHB infection, alone or combined with LC and/or HCC^3^. In the 1980s, vaccination against HBV was introduced in some regions of China^4^. Since 2002 vaccination against HBV has been included in the National Program of Immunizations^5^.

It is believed that the human immune system initiates protective mechanisms following viral infection, including the rapid release of type I interferons (IFNs)^6,7^. Toll-like receptors (TLRs) are the first line of defence against viruses. TLRs stimulate the innate immunity response by recognition of pathogen-associated molecular patterns and promote subsequent adaptive immune responses^8^. Activation of TLR-mediated signaling pathways could inhibit HBV replication^9^, and can also enhance HBV-specific T-cell and B-Cell responses^10^. Of the 10 TLRs in human, TLR3,7,8 and 9 are the important ones against viral infections^11^. Although studies indicated that HBV could not efficiently induce a TLR-mediated immune response, resulting in the lack of type I IFN release in its natural hosts during early phase of infection^12^, the virus can possibly activate TLR pathways in infected hepatocytes and nonparenchymal liver cells to limit viral replication^10^. Previous data indicated that HBV and HBsAg preferentially abrogated TLR9, but not TLR7, agonist-induced IFN-α production in plasmacytoid dendritic cells (pDC)^13^. Lee *et al*. reported that HBV can activate TLR7 with HBcAg packaged single-stranded RNA (ssRNA)^14^. Although the exact interaction between TLR7 and HBV has not been fully understood, some clinical studies have shown that TLR7 agonists can mediate the antiviral effect. Recently, GS-9620, an oral agonist of TLR7 has been found to induce prolonged suppression of HBV in chronically infected chimpanzees^15^.

The TLR7 gene is located at Xp22.2, spanning three exons^16^. It is expressed on intracellular compartments and involved in the regulation of innate immune response via MyD88-dependent proinflammatory signaling cascades. Associations between TLR7 polymorphisms and autoimmune and viral diseases have been recently reported^17–19^. Schott *et al*. first reported that a TLR7 SNP (c.1-120 T > G) protects from advanced inflammation and fibrosis in male patients with chronic hepatitis C virus (HCV) infection^20^. Yue *et al*. recently found that Chinese female patients who carry the TLR7 rs179009 GG genotype and haplotype GCG of rs179009, rs179010 and rs179012 had an increased susceptibility to HCV infection^21^. Wang *et al*. also found that the polymorphism of TLR7 rs179009 might impair immune responses during HCV infection among the Chinese population^22^. Moreover, the TLR7 rs179010 polymorphism was associated with an increased risk for systemic lupus erythematosus (SLE) in Japanese females^23^. The SNP rs2074109 of TLR7 is located in 31 nucleotide upstream of rs179009. The minor allele frequency of rs2074109 is 0.099 in Japanese and 0.069 in Chinese Han populations, highest among all other populations reported in the 1000 genomes study^24^. The effect of this SNP on health and disease has been not reported. Although associations between polymorphisms of TLR7 and HCV infection have been reported, there has been no reported study to investigate association between TLR7 SNPs and the susceptibility and outcome of an HBV infection. Therefore, we aimed to explore the relationship between these three TLR7 polymorphisms and HBV-related liver diseases in the Chinese Han population to gain a better understanding of the role of TLR7 in the development of HBV infection.

## Results

### Clinical information of study subjects

Demographic and clinical parameters of the patients are summarized in Table 1. There were significant differences in terms of gender ratio, age and clinical parameters except for ALT among three groups of patients. All of the patients were positive for HBsAg and anti-HBc antibodies. Levels of AST, AFP, TBIL and DBIL were increased, but ALB levels were decreased significantly with the progression of the disease (all P < 0.001). HBV-DNA copies were significantly decreased from CHB to LC and HCC (P < 0.001). Other indices of patients, such as white blood cell, hemoglobin, blood platelet, creatinine and prothrombin activity were also examined. No increasing/decreasing trends were observed among the three groups of patients.

**Table 1:** Clinical information of studied patients*.

Chracteristics	Chronic hepatitis B	Liver cirrhosis	Hepatocellular carcinoma	P
Patients (n)	250	219	143	
Median age (range, y)	35(12–78)	49(22–86)	53.5(31–77)	<0.001
Gender (male/female)	147/103	148/71	124/19	<0.001
HBeAg (+) (%)	135(54.0)	48(21.9)	24(16.8)	
(male/female)	73/62	31/17	22/2	0.002
HBeAg (−) (%)	79(31.6)	124(56.6)	100(69.9)	
(male/female)	49/30	85/39	87/13	<0.001
HBV-DNA^a^ (copies/mL in log)	3.9(1.3–8.6) (176/250)^b^	3.45(1.2–7.7) (92/219)	3.1(1.4–7.9) (64/143)	0.004
ALT^a^ (U/L)	27.35(3.2–872) (242/250)	28.4(4.8–998.5) (214/219)	32.4(8.7–573.5) (142/143)	0.371
AST^a^ (U/L)	24.8(13.2–707) (242/250)	32.8(6.3–502.8) (214/219)	33.95(12.4–285.2) (142/143)	<0.001
AFP^a^ (mg/L)	2.55(0–365.7) (203/250)	2.955(0.61–447.8) (208/219)	6.93(0.61–807.6) (139/143)	<0.001
TBIL^a^ (µmol/L)	11.6(0.9–711) (242/250)	19(5.9–735.2) (214/219)	18.95(1.6–397.9) (142/143)	<0.001
DBIL^a^ (µmol/L)	2.2(0.1–87.1) (242/250)	4.1(0.9–345.2) (214/219)	4.2(0.8–184.9) (142/143)	<0.001
ALB^a^ (g/L)	44.5(23.3–52.6) (241/250)	41(15.4–51.5) (214/219)	39.9(24.3–52.2) (142/143)	<0.001

^a^indicates the median.

^b^indicates the number of patients whose clinical data available vs total number of patients throughout the table.

*P value of comparison among groups of CHB, LC and HCC by x^2^ test/One-Way ANOVA/nonparametric Kruskal Wallis test.

### Polymorphisms of TLR7 and susceptibility to CHB-related diseases

The observed genotype distributions of these three SNPs were in agreement with the Hardy-Weinberg equilibrium (all P values > 0.05). For TLR7 rs179010, the prevalence of major allele C was significantly higher in male patients with combined CHB, LC and HCC than that of controls (68.3% vs 59.5%, P = 0.029) (Table 2). After subsets stratification, a significantly higher frequency was found in male patients with CHB (P = 0.002) adjusted by age (Table 3). No differencea in distributions of genotypes and alleles were observed between female patients with HBV-related CHB, LC and HCC combined or alone and controls. No significant differences in frequencies TLR7 rs2074109 and rs179009 polymorphisms were observed when male or female patients with CHB, LC and HCC (either combined or alone) were compared with controls.

**Table 2:** The genotypes and allele frequencies of three TLR7 SNPs in patients with CHB, LC and HCC combined (cases) and controls.

Genotypes and allele frequencies	Cases, No (%)	Controls, No (%)	OR(95%CI)	P value
**Male**	No = 419	No = 195		
TLR7 rs179010 major allele C	286(68.3)	116(59.5)	1	Reference
TLR7 rs179010 minor allele T	133(31.7)	79(40.5)	0.818(0.683–0.979)	**0.029**
**Female**	No = 193	No = 98		
TLR7 rs179010 CC	82(42.5)	46(46.9)	1	Reference
TLR7 rs179010 CT	85(44)	45(45.9)	0.944(0.566–1.573)	0.824
TLR7 rs179010 TT	26(13.5)	7(7.1)	0.480(0.193–1.192)	0.108
TLR7 rs179010 major allele C	249(64.5)	137(69.9)	1	Reference
TLR7 rs179010 minor allele T	137(35.5)	59(30.1)	0.783(0.541–1.133)	0.193
**Male**	No = 419	No = 195		
TLR7 rs2074109 major allele T	395(94.3)	185(94.9)	1	Reference
TLR7 rs2074109 minor allele C	24(5.7)	10(5.1)	0.890(0.417–1.899)	0.762
**Female**	No = 193	No = 98		
TLR7 rs2074109 TT	167(86.5)	85(86.7)	1	Reference
TLR7 rs2074109 CT	24(12.4)	13(13.3)	1.064(0.516–2.194)	0.866
TLR7 rs2074109 CC	2(1.1)	0	/	0.553
TLR7 rs2074109 major allele T	358(92.7)	183(93.4)	1	Reference
TLR7 rs2074109 minor allele C	28(7.3)	13(6.6)	0.908(0.459–1.795)	0.782
**Male**	No = 419	No = 195		
TLR7 rs179009 major allele A	347(82.8)	167(85.6)	1	Reference
TLR7 rs179009 minor allele G	72(17.2)	28(14.4)	0.808(0.503–1.298)	0.378
**Female**	No = 193	No = 98		
TLR7 rs179009 AA	130(67.4)	75(76.5)	1	Reference
TLR7 rs179009 AG	56(29)	20(20.4)	0.619(0.345–1.110)	0.106
TLR7 rs179009 GG	7(3.6)	3(3.1)	0.743(0.187–2.959)	0.931
TLR7 rs179009 major allele A	316(81.9)	170(86.7)	1	Reference
TLR7 rs179009 minor allele G	70(18.1)	26(13.3)	0.690(0.424–1.124)	0.135

P value was adjusted for age by logistic regression between cases and controls.

**Table 3:** The genotypes and allele frequencies of TLR7 (rs179010-C/T, rs2074109-T/C and rs179009-A/G) in patients with CHB and controls.

Genotypes and allele frequencies	CHB, No (%)	Controls,No (%)	OR(95%CI)	P value
**Male**	No = 147	No = 195		
TLR7 rs179010 major allele C	110(74.8)	116(59.5)	1	Reference
TLR7 rs179010 minor allele T	37(25.2)	79(40.5)	0.692(0.546–0.877)	**0.002**
**Female**	No = 103	No = 98		
TLR7 rs179010 CC	46(44.7)	46(46.9)	1	Reference
TLR7 rs179010 CT	46(44.7)	45(45.9)	0.978 (0.548–1.746)	0.941
TLR7 rs179010 TT	11(10.6)	7(7.1)	0.636(0.227–1.786)	0.388
TLR7 rs179010 major allele C	138(67)	137(69.9)	1	Reference
TLR7 rs179010 minor allele T	68(33)	59(30.1)	0.874(0.574–1.332)	0.531
**Male**	No = 147	No = 195		
TLR7 rs2074109 major allele T	136(92.5)	185(94.9)	1	Reference
TLR7 rs2074109 minor allele C	11(7.5)	10(5.1)	0.668(0.276–1.619)	0.369
**Female**	No = 103	No = 98		
TLR7 rs2074109 TT	89(86.4)	85(86.7)	1	Reference
TLR7 rs2074109 CT	13(12.6)	13(13.3)	1.047(0.459–2.387)	0.913
TLR7 rs2074109 CC	1(1)	0	1.000	0.553
TLR7 rs2074109 major allele T	134(94.4)	183(93.4)	1	Reference
TLR7 rs2074109 minor allele C	8(5.6)	13(6.6)	0.905(0.419–1.953)	0.798
**Male**	No = 147	No = 195		
TLR7 rs179009 major allele A	128(87.1)	167(85.6)	1	Reference
TLR7 rs179009 minor allele G	19(12.9)	28(14.4)	1.130(0.604–2.113)	0.703
**Female**	No = 103	No = 98		
TLR7 rs179009 AA	70(68)	75(76.5)	1	Reference
TLR7 rs179009 AG	30(29.1)	20(20.4)	0.622(0.324–1.195)	0.153
TLR7 rs179009 GG	3(2.9)	3(3.1)	0.933(0.182–4.778)	1.000
TLR7 rs179009 major allele A	170(82.5)	170(86.7)	1	Reference
TLR7 rs179009 minor allele G	36(17.5)	26(13.3)	0.722(0.418–1.249)	0.243

P value was adjusted for age by logistic regression between patients with CHB and controls.

### Polymorphisms of TLR7 and CHB disease progression

The genotype and allele distributions of the three SNPs of CHB, LC and HCC groups are shown in Table 4.

**Table 4:** Comparison of TLR7 rs179009 distributions among three groups of patients who had confirmed CHB, LC and HCC.

rs179009	CHB, N(%)	LC, N(%)	HCC, N(%)	P^a^	P^b^/OR(95%CI)	P^c^/OR(95%CI)
**Male**	N = 147	N = 148	N = 124			
A	128(87.1)	123(83.1)	96(77.4)	Ref.	Ref.	Ref.
G	19(12.9)	25(16.9)	28(22.6)	**0.012**	0.339/0.730 (0.383–1.393)	**0.029**/2.435 (1.094–5.419)
**Female**	N = 103	N = 71	N = 19			
AA	70(68)	48(67.6)	12(63.2)	Ref.	Ref.	Ref.
AG	30(29.1)	20(28.2)	6(31.6)	0.910	0.935/1.029 (0.524–2.019)	0.777/0.857 (0.294–2.497)
GG	3(2.9)	3(4.2)	1(5.3)	0.540	0.978/0.686 (0.133–3.542)	1.000/0.514 (0.049–5.363)
Allele						
A	170(82.5)	116(81.7)	30(78.9)	Ref.	Ref.	Ref.
G	36(17.5)	26(18.3)	8(21.1)	0.656	0.842/0.945 (0.541–1.649)	0.598/0.794 (0.336–1.874)

^a^P value of nonparametric Kruskal Wallis test among three groups of patients with CHB, LC and HCC.

^b^P value was adjusted for age by logistic regression between patients with LC and CHB.

^c^P value was adjusted for age by logistic regression between patients with HCC and CHB.

An increased frequency of the minor allele G of TLR7 rs179009 was found in men along with severity of CHB-related diseases, being from 12.9% in patients with CHB to 22.6% in patients with HCC (P = 0.012) adjusted by age. No difference was found in the genotype frequency of TLR7 rs2074109 and rs179010 among male patients with CHB-related diseases. In contrast, no difference in frequencies of the three studied SNPs were observed among three groups of female patients.

Furthermore, when patients who were HBeAg-positive were compared among the three groups, the frequency of allele G of TLR7 rs179009 increased from 12.3% in male patients with CHB to 31.8% in those with HCC. However, this was not statistically significant (P = 0.059).

We next performed haplotype analysis. Four major haplotypes CTA, TTA, CTG and CCA were observed (Table 5). In the male group, the frequency of haplotype CTA was markedly higher in patients with CHB (P = 0.000). In contrast, a lower frequency of haplotype TTA was found in patients with CHB than in controls (25.2% vs 40%, P < 0.0001). As for disease progression, the possible influence of TLR7 rs179009 on the development from CHB to HCC in men was confirmed by haplotype CTA and CTG distribution in patients with HCC compared to those with CHB (P < 0.0001 for haplotype CTA; P = 0.003 for haplotype CTG) (Table 6). A significant difference of haplotype CTA distribution was found in men who were HBeAg positive with HCC compared to those with CHB (P < 0.00001) (Supplementary Table 1). No significant difference of haplotype CTA distribution was found in men/women who were HBeAg negative and with HCC compared to those with CHB (Supplementary Table 2).

**Table 5:** Haplotype analysis of TLR7 rs179010(C/T), rs2074109(T/C) and rs179009(A/G) in male controls and patients with CHB, LC and HCC.

Haplotype	Controls (%)	CHB (%)	LC (%)	HCC (%)	P^a^/OR (95% CI)	P^b^/OR (95% CI)	P^c^/OR (95% CI)
CTA	40.5	54.4	43.2	37.1	**0.000**/1.738 (1.280–2.361)	0.507/1.109 (0.816–1.507)	0.361/0.858 (0.619–1.191)
TTA	40	25.2	33.8	37.1	<**0.0001**/0.500 (0.359–0.698)	0.085/0.759 (0.554–1.040)	0.433/0.877 (0.632–1.217)
CTG	13.8	12.9	16.9	22.6	0.707/0.918 (0.588–1.434)	0.283/1.257 (0.827–1.910)	**0.005**/1.804 (1.193–2.729)
CCA	5.1	7.5	6.1	3.2	0.210/1.488 (0.796–2.782)	0.6000.191 (0.619–2.295)	0.247/0.613 (0.266–1.415)

^a^P value calculated between patients with CHB and controls.

^b^P value calculated between patients with LC and controls.

^c^P value calculated between patients with HCC and controls.

**Table 6:** Comparison of haplotype distributions among three groups of patients who had confirmed CHB, LC and HCC.

Haplotype	CHB (%)	LC (%)	HCC (%)	P^a^/OR(95% CI)	P^b^/OR(95% CI)
**Male**					
CTA	54.4	43.2	37.1	**0.007**/0.638(0.461–0.883)	<**0.0001**/0.494(0.350–0.697)
TTA	25.2	33.8	37.1	**0.022**/1.517(1.062–2.167)	**0.003**/1.753(1.213–2.534)
CTG	12.9	16.9	22.6	0.176/1.369(0.867–2.162)	**0.003**/1.965(1.250–3.089)
CCA	7.5	6.1	3.2	0.498/0.801(0.420–1.526)	0.031/0.412(0.180–0.943)
**Female**					
CTA	43.2	39.7	27.3	0.562/0.879(0.569–1.359)	0.067/0.495(0.230–1.063)
TTA	33	36.4	38.4	0.476/1.177(0.751–1.845)	0.516/1.267(0.620–2.591)
CTG	17.5	18.3	17.4	0.808/1.171(0.614–1.871)	0.991/0.995(0.399–2.477)
CCA	6.3	4.7	13.2	0.5300.735(0.281–1.923)	0.138/2.252(0.753–6.735)

^a^P value calculated between patients with LC and CHB.

^b^P value calculated between patients with HCC and CHB.

## Discussion

In this study, we found that genetic variation of TLR7 plays a role in susceptibility to CHB infection and affects disease progression from CHB to HCC in Chinese men. To our knowledge this is the first exploratory study to show the relationship between TLR7 polymorphisms and susceptibility and disease progression of chronic HBV infection in humans.

It has been shown that several agonists of TLRs including TLR7 can inhibit HBV replication, probably as a result of the production of cytokines induced by the TLR signaling^9^. Activation of the TLR7 signaling pathway can facilitate the production of antiviral cytokines, including IFNs, while activation of NF-κB induces secretion of TNFα, interleukin-6(IL-6) and IL-12^25^. As one of the TLRs family members, TLR7 recognizes ssRNA derived from viruses. However, no matter where it came from, whether bacterial, yeast or mammalian, the ssRNA encapsidated within HBcAg did function as a TLR7 ligand at the T and B cell levels with TLR7 knock-out mice verification^14^, indicating HBcAg packaged ssRNA could activate TLR7 signaling pathway in HBV natural infection.

Previous *ex vivo* and *in vitro* studies showed that HBV infection could suppress the intracellular TLR-induced antiviral activity of pDCs and liver cells^26,27^. TLR7 is one of such TLRs. Although pDCs display an impaired ability to secrete IFN-α following *ex vivo* stimulation with TLR9/TLR7 ligands during a chronic HBV infection, HBV particle internalization could inhibit TLR9- but not TLR7-mediated secretion of IFN-α by pDCs^13^.

In this study, we assessed the impact of three SNPs of TLR7 on susceptibility to and disease progression of CHB infection in Chinese adults. The three polymorphisms rs179010, rs2074109 and rs179009 with the respective change from C to T, T to C and A to G, do not cause alteration in amino acids. TLR7 rs2074109 is near to rs179009, and located at 31 bp upstream of rs179009. Based on SNP function prediction (http:snpinfo.niehs.nih.gov), mutations of TLR7 rs179009 and 179010 are predicted to occur at transcription factor binding sites (TFBSs), which could affect level and/or timing of TLR7 expression and result in difference in production of downstream cytokines such as IFN-α, suggesting that these SNPs are functional.

Xiao *et al*. reported that the risk of Grave’s Disease (GD) decreased significantly as the frequency of TLR7 rs179010 T alleles increased in Chinese females, indicating a protective effect of TLR7 rs179010 polymorphism against GD^18^. Moreover, this SNP rs179010 was also associated with increased risk for SLE in Japanese females^23^. It has been considered to be associated with autoantibody production, suggesting that TLR7 might increase B-cell sensitivity to RNA-containing autoantigens in the development of systemic autoimmunity^28^. Numerous antagonists targeting the TLR signaling cascade are identified as potential therapeutic targets for SLE^29^, including TLR7 antagonists which could inhibit Th1-type cytokines release, such as IFN-α and modulate the imbalance of Th1/Th2 cytokines environment in SLE patients. Stimulation of TLR7 could mediate an effective Th1-dependent immune response, which is known to be critical in development of a broad and effective protection against hepatitis viruses^25^. Nonetheless, cytokines are the primary cause of inflammation and can mediate liver injury after HBV infection^30^.

In this present study, Chinese men with CHB had a significantly higher frequency in major allele C of TLR7 rs179010, suggesting an increased risk of CHB infection. The finding highlighted that polymorphism of TLR7 rs179010 is functional and its effect may be partially mediated via B cell response during HBV persistent infection. However, in a recent study on the relationship of TLR7 polymorphisms with susceptibility to HCV infection, no association between TLR7 rs179010 and HCV infection was found^21^. It has been shown that TLR7 can recognize HCV RNAs, leading to the production of IFN and antiviral cytokines to influence HCV infection and progression^31^. Recently genetic variation of TLR7 was reported to influence HCV infection with gender difference^32^. In a study conducted in Taiwan, SNP rs179009 G allele of TLR7 was present at a higher frequency in males with HCV infection, as compared to healthy controls^22^.

Interestingly, we found an increased frequency of the minor allele G of TLR7 rs179009 in men along with the severity of CHB-related diseases, indicating an increased risk for development from CHB to HCC. Haplotype analysis also supported these findings, as CTG was confirmed as a risk factor of disease progression for men. Although the pathogenesis of HCV infection differs from that of an HBV infection, TLR7 rs179009 was observed to be related to both of them, which may be due to the impaired host antiviral response. HBeAg+ represents a surrogate of viral replication and indicates an increased susceptibility to HCC^33^. Data showed that HBV virions or elements including HBeAg or HBsAg can suppress TLR7-induced antiviral activity of murine parenchymal and nonparenchymal liver cells^27^. We evaluated the influence of TLR7 rs179009 on disease development among patients of HBeAg(+) and observed that among the male individuals of HBeAg(+), the frequency of allele A was decreased from 87.7% in patients with CHB to 68.2% in those with HCC. However, this change did not reach a statistical significance.

It has been recently shown that variation in the copy number of TLR7 is linked to the disease progression of HBV infection in Chinese population^34^. A low copy number of TLR7 was significantly associated with an increased risk of chronic HBV infection in female patients (P < 0.001). However, no significant differences were found in the copy number of TLR7 among patients with CHB, LC or HCC.

Gender is one of the major factors that influence the outcome and severity of an HBV infection in clinical practice^35^. In line with this, we noticed a gender difference in association of TLR7 rs179010 and 179009 polymorphisms with CHB-related diseases among Chinese men but not in women. The finding might explain at least in part the higher incidence and more severe manifestations of HBV infection in Chinese men compared to women^35,36^. Mounting data has highlighted the sex-based differences in the pathogenesis of infectious and autoimmune diseases, which might attribute to the action of sex hormones and X chromosome-linked genes^37^. Berghöfer *et al*. reported a sex-dependent pathway of TLR7-induced IFN-α with high production in females unrelated to hormonal effects^38^. Meier *et al*. also showed that sex differences in TLR7-mediated activation of pDCs with enhanced activation of CD8+ T cells could account for a higher immune response in women compared to that of men when the same amount of HIV-1 RNA was used. This enhanced immune activation in women might explain the finding on why higher viral loads occur in men during early phase of HIV infection and the clinical observation on why women had a higher risk for HIV-1 disease progression during chronic infection at a given HIV-1 viral load^39^.

The SNP rs2074109 of TLR7 is close to rs179009. The reported frequency of minor allele C based on the 1000 genomes project was only 0.069 in Chinese Han population^24^. In this study, similar minor allele frequencies in both controls and patients were noticed. Moreover, no significant association was found among patients with CHB, LC and HCC, suggesting that this polymorphism may be not functional.

There are certain limitations in this study. The first was a relatively small number of subjects included in each group, especially the number of female patients with HCC. Therefore, a risk of type-2 statistical error may be present due to the low number of female patients with HCC, which means that further studies with a large number of subjects are needed. Secondly, it is known that there are multiple phases of disease in CHB: immune tolerance, immune clearance, immune control and immune escape^40^. These phases are divided based on viral load, ALT, HBeAg and HBeAb. In this present study, all CHB phases were aggregated and analyzed together. Therefore the results should be interpreted with caution as these above-mentioned confounding factors were not taken into analysis. Thirdly, only three intronic SNPs of TLR7 were assessed. Fourth, the function of SNP rs179010 and rs179009 on TLR7 expression and its signaling pathway was not assessed. Some important cytokines like IFN-α and IL-6 were not determined in study subjects.

In conclusion, our findings supported the role of TLR7 rs179010 in predisposition of CHB in Chinese men, while TLR7 rs179009 A allele was associated with a decreased risk of disease progression from CHB to HCC. To our knowledge, this is the first report of TLR7 SNPs and HBV infection. However, given the limited sample size, this finding requires verification by larger studies in diverse ethnicities.

## Methods

### Study subjects

A total of 905 study subjects included 612 patients with confirmed HBV infection recruited from July 2014 to September 2015 in Beijing You’an Hospital, and 293 controls who attended annual physical examination. All study subjects were Han ethnic. The patients were divided into three groups: 250 patients with CHB, 219 patients with LC and 143 patients with HCC (Table 1). The diagnosis of CHB infection was made based on previous history of hepatitis B or positive HBsAg more than 6 months. Serum HBsAg and/or HBV DNA has been positive, and alanine aminotransferase (ALT) or aspartate aminotransferase(AST) intermittent elevated, or liver tissue examination with hepatitis lesions. LC was diagnosed on the basis of a clear previous history of chronic hepatitis B, HBsAg test positive, pathologic exams, laboratory features, and the findings of computed tomography (CT) or ultrasonography. HCC was diagnosed by at least one positive iconography examination result, including CT and magnetic resonance imaging, or positive findings on cytological or pathological examination. All patients were free of other viral infections, including human immunodeficiency virus (HIV), HCV, cytomegalovirus and Epstein–Barr virus. They did not report any other type of liver disease (for example, autoimmune hepatitis, toxic hepatitis, and so on), or other cancers. Clinical samples were collected from those who had not received antiviral treatment or immunotherapy during the past 6 months and if patients had not received any surgical treatment. According to the characteristics of specific serology, patients were divided into HBsAg-, HBeAg-and anti-HBc antibodies positive (here referring as HBeAg(+)) group and HBsAg-, anti-HBe and anti-HBc antibodies positive (here referring as HBeAg(−)) group. Demographical data of all study subjects were collected at the first time of visit to the hospital and the following laboratory parameters were obtained and were available for most of the patients, such as serum AST, ALT, Alphafetoprotein(AFP), total bilirubin(TBIL), direct bilirubin(DBIL), albumin (ALB) and HBV-DNA copies (Table 1). The median and age range of patients (N = 612) and controls (N = 293) were 45 years (12–86 years) and 39 years (21–60 years), respectively. No significant difference was found in gender ratio between patients (male vs female: 419/193) and controls (195/98).

This study was approved by the Ethics Committee of Beijing You’an Hospital, Capital Medical University. Informed consent for both study participation and publication of identifying information/images (when applicable) was obtained from each of the participants at the time of inclusion. The methods were carried out in accordance with the relevant guidelines, including any relevant details.

### TLR genotyping

Genomic DNA was extracted from blood using a commercial kit according to the manufacturer’s instructions (QIAGEN DNA Blood Mini Kit, Hilden, Germany). Three SNPs (rs179010, rs2074109 and rs179009) of the TLR7 gene were identified using PCR-based sequencing. Primers for TLR7 rs179010 were forward (5′-AGCCAGTCCACGGTTAAAGC3′) and reverse (5′-AGCCCAAGGTTACCCAGTAG3′) and primers for TLR7 rs2074109 and rs179009 were forward (5′-AGCAGGCCGACATAAATTGC3′) and reverse (5′-GTCTGTGCAGTCCACGATCA3′). PCR was performed in a total reaction volume of 50 µl with 100–200 ng genomic DNA. After an initial denaturation at 95 °C for 2 min, the DNA was amplified for 38 cycles at 95 °C for 30 s, 60 °C for 30 s, and 72 °C for 1 min 10 s, with a final elongation at 72 °C for 5 min on the PCR System 9700 (PE Applied Biosystems, Foster City, CA, USA). Both positive and negative controls were used in each PCR run. The amplicons were sequenced at the Sino Geno Max Co., Ltd, Beijing, China and SNPs were identified by the computer program of mutation surveyor V5.0.0 (SoftGenetics, USA). To validate the sequencing results obtained by the forward sequencing primer, every tenth of the PCR-amplified DNA samples were selected and re-sequenced by the reverse primer. The results obtained between the two sequencing analyses were completely concordant.

### Statistical analysis

For each SNP, allele and genotype frequencies were descriptively summarized. Statistical analysis was carried out using SPSS statistical software version 17.0 (SPSS Inc., Chicago, USA). Because the TLR7 gene is located on X chromosome, the allele frequency of each SNP was separately analyzed for male and female patients. Each SNP was tested for deviation from Hardy-Weinberg equilibrium (HWE). Differences of demographic data, genotype, allele frequencies between cases and controls or among three groups of patients (CHB, LC and HCC) were evaluated by Student’s t-test, chi-square test, one-way analysis of variance (ANOVA) or nonparametric test and Kruskal-Wallis test where appropriate. Logistic regression was used for the multivariate analyses throughout the paper, adjusted for age. P values, odds ratios (ORs), and 95% confidence intervals (95% CIs) were used to evaluate the association between polymorphisms and the risk of disease. SHESIS on line (http://analysis.bio-x.cn/myAnalysis.php) were used for haplotype analysis. A two-sided P value of less than 0.05 was considered significant.

## Electronic supplementary material

Supplemenatary tables
