# Supplementary material for: Toll like receptor7 polymorphisms in relation to disease susceptibility and progression in Chinese patients with chronic HBV infection
Source: Sci Rep. 2017 Sep 29;7:12417. doi: 10.1038/s41598-017-12698-5 (PMC5622102; doi:10.1038/s41598-017-12698-5)
Supplement: Supplementary file 1 — Supplemenatary tables [file 41598_2017_12698_MOESM1_ESM.pdf]

Toll like receptor7 polymorphisms in relation to disease susceptibility and progression in Chinese patients with chronic HBV infection

Junping Zhu [1,a], Tong Zhang [2,a], Lina Cao [1], Aixin Li [2], Kai Zheng [1], Nan Zhang [1], Bin Su [2], Zhiyun Chen[1], Ning Chen [1], Hao Wu* [2], Qiushui He* [1,3]

Supplementary materials

Table 1. Comparison of haplotype distributions among three groups of patients who had confirmed CHB, LC and HCC and who were HBeAg(+)

Haplotype	CHB (%)	LC (%)	HCC (%)	P*/OR(95% CI)	P‡/OR(95% CI)	P†/OR(95% CI)
**Male**						
CTA	55.4	51.6	17.4	0.615/0.859 (0.474-1.555)	**0.000**/0.197 (0.079-0.491)	**<0.00001**/0.169 (0.074-0.388)
TTA	27	29	39.1	0.767/1.105 (0.572-2.132)	0.271/1.571 (0.701-3.522)	0.117/1.736 (0.867-3.476)
CTG	12.2	19.4	30.4	0.174/1.733 (0.779-3.857)	0.183/1.823 (0.749-4.437)	**0.004**/3.160 (1.422-7.021)
CCA	5.4	0	13	0.062	**0.003**	0.080/2.625 (0.861-8.008)
**Female**						
CTA	51.6	55.9	/	0.655/1.190 (0.555-2.552)	/	/
TTA	25.8	20.5	/	0.529/0.744	/	/

				(0.295-1.874)		
CTG	15.3	14.7	/	0.931/0.954	/	/
				(0.328-2.775)		
CCA	7.2	8.8	/	0.764/1.233	/	/
				(0.314-4.842)		

*P value calculated between patients with LC and CHB.

‡P value calculated between patients with HCC and LC.

†P value calculated between patients with HCC and CHB.

Table 2. Comparison of haplotype distributions among three groups of patients who had confirmed CHB, LC and HCC and who were HBeAg(-)

Haplotype	CHB (%)	LC (%)	HCC (%)	P*/OR(95% CI)	P‡/OR(95% CI)	P†/OR(95% CI)
**Male**						
CTA	46.9	39.8	42.5	0.250/0.747 (0.453-1.229)	0.600/1.121 (0.732-1.716)	0.482/0.837 (0.509-1.376)
TTA	30.6	33	37.9	0.691/1.114 (0.654-1.897)	0.330/1.243 (0.802-1.928)	0.225/1.385 (0.817-2.348)
CTG	14.3	18.2	18.4	0.408/1.333 (0.673-2.640)	0.960/1.014 (0.590-1.744)	0.386/1.352 (0.683-2.678)
CCA	8.2	9.1	1.1	0.795/1.125 (0.463-2.732)	**0.000**/0.116 (0.026-0.514)	**0.003**/0.131 (0.027-0.629)
**Female**						
CTA	35.5	34.6	32.1	0.956/0.980 (0.487-1.974)	0.780/0.877 (0.349-2.203)	0.755/0.860 (0.333-2.220)
TTA	41.9	46.2	35.7	0.569/1.216 (0.620-2.388)	0.313/0.633 (0.259-1.546)	0.578/0.770 (0.306-1.937)
CTG	19.4	15.4	14.3	0.558/0.769 (0.319-1.856)	0.870/0.903 (0.265-3.072)	0.560/0.694 (0.203-2.380)
CCA	3.2	2.6	17.9	0.832/0.806 (0.110-5.916)	**0.006**/8.151 (1.482-44.849)	**0.016**/6.572 (1.185-36.455)

*P value calculated between patients with LC and CHB.

‡P value calculated between patients with HCC and LC.

†P value calculated between patients with HCC and CHB.
